# Development of *Onchocerca volvulus* in humanized NSG mice and detection of parasite biomarkers in urine and serum

**DOI:** 10.1371/journal.pntd.0006977

**Published:** 2018-12-12

**Authors:** John B. Patton, Sasisekhar Bennuru, Mark L. Eberhard, Jessica A. Hess, April Torigian, Sara Lustigman, Thomas B. Nutman, David Abraham

**Affiliations:** 1 Department of Microbiology and Immunology, Thomas Jefferson University, Philadelphia Pennsylvania, United States of America; 2 Laboratory of Parasitic Diseases, National Institute of Allergy and Infectious Diseases, Bethesda, Maryland, United States of America; 3 Division of Parasitic Diseases and Malaria, CDC, Atlanta, Georgia, United States of America; 4 Laboratory of Molecular Parasitology, Lindsley F. Kimball Research Institute, New York Blood Center, New York, New York, United States of America; University of Zurich, SWITZERLAND

## Abstract

**Background:**

The study of *Onchocerca volvulus* has been limited by its host range, with only humans and non-human primates shown to be susceptible to the full life cycle infection. Small animal models that support the development of adult parasites have not been identified.

**Methodology/Principal findings:**

We hypothesized that highly immunodeficient NSG mice would support the survival and maturation of *O*. *volvulus* and alteration of the host microenvironment through the addition of various human cells and tissues would further enhance the level of parasite maturation. NSG mice were humanized with: (1) umbilical cord derived CD34^+^ stem cells, (2) fetal derived liver, thymus and CD34^+^ stem cells or (3) primary human skeletal muscle cells. NSG and humanized NSG mice were infected with 100 *O*. *volvulus* infective larvae (L3) for 4 to 12 weeks. When necropsies of infected animals were performed, it was observed that parasites survived and developed throughout the infection time course. In each of the different humanized mouse models, worms matured from L3 to advanced fourth stage larvae, with both male and female organ development. In addition, worms increased in length by up to 4-fold. Serum and urine, collected from humanized mice for identification of potential biomarkers of infection, allowed for the identification of 10 *O*. *volvulus*-derived proteins found specifically in either the urine or the serum of the humanized *O*. *volvulus*-infected NSG mice.

**Conclusions/Significance:**

The newly identified mouse models for onchocerciasis will enable the development of *O*. *volvulus* specific biomarkers, screening for new therapeutic approaches and potentially studying the human immune response to infection with *O*. *volvulus*.

## Author summary

Onchocerciasis, caused by the filarial parasitic nematode *Onchocerca volvulus*, remains a significant source of morbidity throughout sub-Saharan Africa and is a primary cause of infectious blindness. Research on this disease has been hindered by an absence of suitable small animal hosts. Here we describe the development of humanized mouse models that are susceptible to *O*. *volvulus* infection supporting both growth and maturation of the parasite. These novel mouse models have enabled the identification of a number of new *O*. *volvulus* biomarkers with potential to play a role in the development of specific and sensitive diagnostic tests for presence of viable parasites.

## Introduction

Onchocerciasis, caused by the parasitic filarial nematode *Onchocerca volvulus*, remains a significant source of morbidity throughout sub-Saharan Africa [1]. *O. volvulus* infection is commonly diagnosed through the presence of microfilariae in skin snips, but skin samples can be further analyzed by qPCR for enhanced sensitivity [2]. Antibody tests (e.g. Ov16 [3,4]) are available but do not have the ability to differentiate between past and present infections which is problematic in areas where the infection is endemic [5]. Recently, a limited number of biomarkers have been identified in the urine that can distinguish between *O*. *volvulus*-infected and non-infected individuals [6–8].

A significant obstacle for studying the biology of *O*. *volvulus* and for the development of new therapeutics and diagnostics has been the absence of small animal models. The only susceptible animal hosts for *O*. *volvulus* are chimpanzees [9,10] and mangabey monkeys [11]. Chimpanzees infected with *O*. *volvulus* had patent infections that lasted between 6 to 9 years with adult-worm bundles located in deep tissues with microfilariae in skin snips being detected 12–18 months post infection [12,13]. While immunologically intact mice are resistant to infection with the infective larvae (L3) of *O*. *volvulus* [14], adult worms within nodules have been successfully transplanted into SCID mice (NOD.CB17-*Prkdc*^*scid*^/J) with the worms surviving greater than 20 weeks [15]. This observation was confirmed by the successful transplantation of adult *Onchocerca ochengi* into SCID mice [16]. As an alternative approach, *O*. *volvulus* L3 were implanted in primates and rodents within diffusion chambers that consist of a Lucite ring enclosed with permeable membranes, allowing for migration of cells and other humoral factors into the diffusion chamber with simultaneous containment of the parasites. *O*. *volvulus* in diffusion chambers implanted in multiple species of rodents and primates showed limited growth of larvae in all host species [17].

Several strategies have been employed to overcome murine resistance to infection with various human pathogens. The Collaborative Cross (CC) is a large group of inbred mouse strains that were developed to address many of the different shortcomings found within the existing experimental mouse populations, including small numbers of homozygous strains, limited genetic diversity, and non-ideal population structures. Based on the hypothesis that there is a genetic basis for mouse susceptibility and resistance to infection, novel strains of CC mice have been identified that are susceptible to specific bacteria, viruses, and parasites of humans [18–23].

As an alternative approach to overcome murine resistance to infection, mice have been developed with greatly diminished immune responses. SCID mice were shown to be susceptible to infection with *Brugia malayi* while immunocompetent mice were resistant to the infection [24]. NOD.Cg-*Prkdc*^*scid*^*Il2rg*^*tm1Wjl*^/SzJ (NSG) mice are a highly immune-compromised strain of mice that have profound defects in the adaptive and innate immune responses [25]. The most notable defects include those in: 1) macrophages, dendritic cells and in the complement cascade [26–28]; 2) maturation of T and B cells [29]; 3) NK cells; 4) signaling of 6 different cytokines [30] and 5) the presence of eosinophils in the peripheral circulation and in tissue [31]. NOD-*Rag1*^*tm1Mom*^ *IL2rg*^*tm1Wjl*^ mice (NRG) are phenotypically similar to NSG mice with disruption in the B and T-Cell production [32]. Interestingly, NSG mice can support the complete lifecycle of the human nematode *Strongyloides stercoralis*, whereas immunologically intact mice cannot [31].

A third strategy to enhance pathogen survival in mice has been to add human-derived cells required for survival and growth [33]. NSG mice have the unique ability to support several different xenografts including human hematopoietic stem cells (CD34^+^ stem cells) that allow for the development of an immature partially-functional human immune system [25,27,30,34]. NOD.Cg-*Prkdc*^*scid*^ *Il2rg*^*tm1Wjl*^ Tg (CMV-IL3, CSF2, KITLG)1Eav/MloySzJ (SGM) mice have an additional three human genes IL3, CSF2, and KITLG under the CMV promoter to enhance the overall microenvironment for the development of human xenografts. This results in increased numbers of CD33^+^ myeloid cells, B-cells, T-cells, and hematopoietic stem cells [35]. Humanized BLT mice are NSG mice that received a xenograft of human CD34^+^ stem cells and a transplant of human fetal thymus and liver implanted under the kidney capsule, which results in the formation of a “human immune organ” [36].

Control and potential elimination of onchocerciasis has been significantly impeded by the limited number of available drugs and the development of resistance to those therapies [37,38]. In addition, biomarkers to assess the infection status of treated individuals or macrofilaricidal activity are sorely lacking [6–8]. One of the critical barriers blocking drug and biomarker development has been the absence of suitable small animal hosts for experimentation. Hence, this study was focused on the development of a small animal model that would support the growth and maturation of *O*. *volvulus* and could serve to identify parasite specific biomarkers. To this end we tested multiple genetically defined mouse strains and xenografted (with human cells) immunodeficient mice to identify those microenvironments suitable for *O*. *volvulus* development. In so doing, we were able to develop convenient and tractable murine models that support the development of *O*. *volvulus* L3 into advanced larval stages. Moreover, we were able to use these *O*. *volvulus*-infected animals to identify parasite-derived biomarkers measurable in both urine and serum.

## Materials and methods

### Ethics statement

The parasite material was collected during the years 1994–1999 in the research facility at the Tropical Medicine Research Station, Kumba, Cameroon. The procedures used for the production of *O*. *volvulus* forest strain third-stage-larvae (L3) were approved by an NIH accredited Institutional Review Board of the Medical Research Council Kumba, Cameroon (Protocol 001). The protocol was reviewed and approved annually. L3 were collected from black flies (*Simulium damnosum*) that were fed on consenting infected donors. After seven days the flies were dissected and the developed L3 were collected, cleaned and cryopreserved. The cryopreserved L3 were shipped to the New York Blood Center in liquid nitrogen and upon arrival in New York were stored in liquid nitrogen. All protocols using the L3 cryopreserved samples in this study were approved by the New York Blood Center's IRB (Protocol 321 and Protocol 603–09). All L3 samples were anonymized.

All experimental procedures in mice were performed in compliance with the ethical and regulatory standards set by the NIH for animal experimentation. The animal use protocol (01469) was approved by the Thomas Jefferson University Institutional Animal Care and Use Committee. The animal care and use protocol adhered to the “Guide for the Care and Use of Laboratory Animals” published by the National Research Council, USA.

### Parasites and infection of mice

Cryopreserved L3 were prepared as previously described [39–41]. Briefly, black flies (*Simulium damnosum*) were fed on consenting donors infected with *O*. *volvulus*, and after 7 days the developed L3 were collected from dissected flies, cleaned, and cryopreserved in dimethyl sulfoxide and sucrose using Biocool II computerized freezing equipment (FTS Systems Inc., Stone Ridge, NY) [42].

Cryopreserved L3 were removed from liquid nitrogen storage and placed on dry ice for 15 minutes followed immediately by a 37° water bath. The L3 were then washed 5 times in a 1:1 mixture of NCTC-135 and Iscove’s modified Dulbecco’s medium (Sigma, St. Louis MO) supplemented with 100 U penicillin, 100 μg streptomycin (Corning, Tewksbury MA) 100 μg gentamicin and 30 μg chloramphenicol per ml (Sigma). L3 isolated from different collection days were tested first for viability in diffusion chambers implanted in BALB/ByJ for 21 days, as previously described [17]. Batches of L3 with viabilities greater than 50% at 21 days post implantation were used in these studies. One hundred worms (except where noted) were then counted and loaded into 1 ml tuberculin syringes with 21 g needle for subcutaneous injection of the larvae into the nape of the neck.

### Mice

All mice were housed in micro-isolator boxes in a pathogen-free room at the Laboratory Animal Science Facility at Thomas Jefferson University (Philadelphia, PA). Collaborative Cross (CC) mouse strains, Cast/EIJ, IL16680 (CC055/TauUnc), AU8052 (CC052/GeniUnc), AU8049 (CC038/GeniUnc) and OR13067 (CC003/Unc), were purchased and imported from the Systems Genetics Core Facility of University of North Carolina (UNC-Chapel Hill). Information about the CC strains can be found on the UNC Systems Genetics website at http://csbio.unc.edu/CCstatus/index.py. The mice were kept under temperature, humidity and light cycle-controlled conditions and fed autoclavable rodent chow and given water *ad libitum*.

NOD-*scid* IL2Rg^null^ (NSG), NOD-*Rag1*^*null*^ *IL2rg*^*null*^ (NRG), and NOD-*scid* IL2Rg^null^-3/GM/SF (SGM) mice were obtained from The Jackson Laboratories (Bar Harbor, ME). An NSG, NRG, and SGM mouse breeding colony was maintained in the Laboratory Animal Science Facility, at Thomas Jefferson University with breeding trios given acidified water and low fat 5K52 animal chow (LabDiet, St. Louis, MO).

### Injection of cells for single cell xenografts

The following human cell types were individually transferred into NSG mice: (1) human keratinocytes (HaCat) (ATCC, Manassas, VA), (2) bovine embryo skeletal muscle cells (BESM) [43], (3) lymphatic endothelial cells (LEC) (PromoCell, Heidelberg, Germany), and (4) human skeletal smooth muscle cells (HuSkMc) (Cell Application, San Diego, CA). HaCat and BESM were maintained in Dulbecco’s Modification of Eagle’s Medium (DMEM) (Corning, Manassas, VA) supplemented with 100 U penicillin, 100 μg streptomycin (Corning), 200 nM L-glutamine (Corning) and 10% fetal bovine serum (FBS) (Gemini BioProducts, West Scaramento, CA). LEC were maintained in EGM-2MV media (Lonza, Walkersville, MD), and HuSkMc were maintained in Skeletal Muscle Growth Medium (Cell Applications Inc, San Diego, CA) following manufacturer’s recommendations.

In the initial experiments, mice were injected with 5×10^6^ BESM, HaCaT, LEC, or HuSkMc cells subcutaneously weekly throughout the experiment. The frequency of injection was subsequently determined by *in vivo* imaging experiments. Genes encoding green fluorescent protein (GFP) and luciferase were inserted into HuSkMc cells using lentiviral vectors following the manufacturer’s recommendations (Cell Biolabs, Inc, San Diego, CA). Cells expressing GFP were isolated using the GFP marker by fluorescence-activated cell sorting using a BD FACS Aria (BD Biosciences, Franklin Lakes, NJ). The isolated cells were grown in HuSkMc media as described above and 5×10^6^ cells were injected subcutaneously into NSG mice. Mice were injected with VivoGlo (Promega, Madison, WI) and imaged following manufacturer’s recommendations on an IVIS Lumina XR (Promega).

### Humanized mice with multiple human cell types

Two approaches were used to create humanized mice containing multiple human-cell types [25,36]. Human umbilical cord blood was obtained through collaboration with Thomas Jefferson University Hospital Department of Obstetrics and Gynecology from full term natural deliveries. CD34^+^ stem cells were isolated from cord blood using magnetic assisted cell sorting (MACS) and cryopreserved until use. SGM, NRG and NSG mice were humanized with CD34^+^ umbilical cord derived stem cells by intrahepatic injection of 5x10^5^ CD34^+^ stem cells into 48-hour old pups that were irradiated with 1.5 gray. Six weeks following injection of the stem cells, peripheral blood from the mice was screened for the presence of human cells and mice with counts greater than 600 human CD45^+^ hematopoietic cells per μl of whole blood were used for experimentation, following previously published protocols [31].

BLT mice were purchased from The Jackson Laboratories or were prepared following previously established protocols [27]. Briefly, NSG mice (4- to 6-week old) were implanted with 1 mm^3^ sections of fetal thymus and liver (Advanced Biomedical Resources, Alameda, CA) under the kidney capsule. Two weeks post-implantation the mice were treated with busulfan (Sigma) (20 mg/kg IP) and were injected retro-orbitally with 5x10^5^ CD34^+^ stem cells, isolated from the donor fetal liver using MACS, (Miltenyi Biotec Inc. Auburn, CA). Eight weeks following the stem cell xenograft the peripheral blood from the BLT mice was screened for the presence of human cells. BLT mice were screened and selected using the same protocol described above for the CD34^+^ cord blood mice.

### qPCR screening of tissues for the presence of *O*. *volvulus* parasites

PCR screening for *O*. *volvulus* DNA was performed on all the infected mouse tissues to identify the presence of current or past *O*. *volvulus* larvae in that location. Mice were anesthetized and exsanguinated, and the internal organs were removed, and the skin was removed from the muscle. The muscle and skin were then divided into 100 different sections and individually frozen in 1.7 ml Eppendorf tubes. DNA was extracted from the tissue sections using the Promega genomic DNA kit A1125 following the manufacturer’s directions. Realtime PCR was performed using custom Taqman probes (Integrated DNA Technologies, Coralville, IA) against the Ov-150 [44–46] repeats and an ABI OneStep-Plus (ThermoFisher).

### Necropsy and morphological analysis

Mice were necropsied following previously established protocols for the isolation of filarial worms from tissues [47,48]. Briefly, mice were anesthetized using isoflurane gas and exsanguinated. The head was removed from the body of the mouse and discarded. The remaining internal organs and skin were removed from the muscles, and the muscle was divided into upper and lower sections at the bottom of the rib cage. All portions of the mouse (muscle, skin, and all internal organs with the exception of the head) were soaked overnight in RPMI containing 10% FBS and with 100 U penicillin, 100 μg streptomycin (Corning), and emerging parasites were then collected and enumerated. Infected mice were evaluated using two criteria: 1) percent established, measured the proportion of mice in a group of infected animals from which live parasites were recovered; and 2) the geometric mean number of live worms recovered per mouse within the group.

Recovered worms were placed in boiling fixative consisting of 95% ethanol (Deacon Labs, King of Prussia, PA) and 5% glycerol (Fisher, Fair Lawn, NJ). After allowing the alcohol to evaporate, glycerol was added, and the worms were transferred to glycerin jelly (gelatin 10 g, ddH_2_O 60.0 ml, glycerine 70.0 ml, Phenol 1.0 ml). Fixed worms were measured using an Olympus SZX16 dissecting scope connected to a DP26 camera (Olympus, Center Valley, PA). CellSens Dimensions software (Olympus) was used to measure the length of the recovered worms.

### Serum and urine proteomics

Serum and urine were collected and frozen as terminal procedures during necropsy. Serum was thawed on ice and 25 μL removed for processing from each mouse. Sera from 4 mice from each group/strain were pooled for maximizing the protein identifications. Abundant proteins were depleted using an affinity chromatography (MARS-Ms-3, Agilent) according to the manufacturer’s directions. Urine was thawed on ice, centrifuged and then filtered through a 0.22 μM filter (Corning). Serum and urine samples were prepared for mass spectrometry by digestion using the filter-assisted sample preparation (FASP) method [49]. Briefly, the samples brought to 1% sodium deoxycholate (SDC), 50 mM Tris-HCl, pH 7.6, 3 mM dithiothreitol, sonicated briefly, and incubated in a Thermo-Mixer at 90^o^ C, 1,000 RPM for 20 min. Samples were centrifuged to clarify and the supernatant was transferred to a passivated 30 kD MWCO device (Millipore, Merck KGaA, Darmstadt, Germany) and centrifuged at 13,000g for 30 min. The remaining sample was buffer exchanged with 1% SDC, 100 mM Tris-HCl, pH 7.6, then alkylated with 15 mM iodoacetamide. The SDC concentration was reduced to 0.1%. Samples were digested using trypsin at an enzyme to substrate ratio of 1:100, overnight, at 37^o^ C in a thermo-mixer at 1,000 RPM. Digested peptides were collected by centrifugation and the filter washed with 0.5 NaCl to elute electrostatically bound peptides. Digested peptides were desalted using reversed phase stop-and-go extraction tips [50]. Peptides were eluted with 80% acetonitrile, 0.5% formic acid and lyophilized in a SpeedVac (Thermo Savant, Holbrook, NY) to near dryness, approximately 1 h.

### Liquid chromatography-tandem mass spectrometry

Each digestion mixture was analyzed by ultra-high performance liquid chromatography tandem mass spectrometry (UHPLC-MS/MS). LC was performed using an Easy-nLC 1000 UHPLC system (Thermo Fisher Scientific, Waltham, MA). Mobile phase A was 97.5% MilliQ water, 2% acetonitrile, 0.5% formic acid. Mobile phase B was 99.5% acetonitrile, 0.5% formic acid. The 240 min LC gradient ran from 0% B to 35% B over 210 min, then to 80% B for the remaining 30 min. Samples were loaded directly to the column. The column was 50 cm x 75 um I.D. and packed with 2 μm C18 media (Thermo Easy Spray PepMap). The LC was interfaced to a quadrupole-Orbitrap mass spectrometer (Q-Exactive, Thermo Fisher Scientific, Waltham, MA) via nano-electrospray ionization using a source with an integrated column heater (Thermo Easy Spray, Thermo Fisher Scientific, Waltham, MA). The column was heated to 50°C. An electrospray voltage of 2.2 kV was applied. The mass spectrometer was programmed to acquire, by data-dependent acquisition, tandem mass spectra from the top 10 ions in the full scan from 400–1200 m/z. Dynamic exclusion was set to 15 s, singly-charged ions were excluded, isolation width was set to 1.6 Da, full MS resolution to 70,000 and MS/MS resolution to 17,500. Normalized collision energy was set to 25, automatic gain control to 2e5, max fill MS to 20 ms, max fill MS/MS to 60 ms and the underfill ratio to 0.1%.

### Mass spectroscopy data processing and library searching

Mass spectrometer RAW data files were converted to mzML format using msconvert [51]. MGF files were generated from mzML using the Peak Picker HiRes tool, part of the OpenMS framework [52]. All searches were performed on Amazon Web Services-based cluster compute instances using the Proteome Cluster interface. Detailed search parameters are printed in the search output XML files. Briefly, all searches required 10 ppm precursor mass tolerance, 0.02 Da fragment mass tolerance, strict tryptic cleavage, up to 2 missed cleavages, fixed modification of cysteine alkylation, variable modification of methionine oxidation and protein-level expectation value scores of 0.0001 or lower. Proteome Cluster builds species- and genus-specific protein sequence libraries from the most current UniProtKB distribution [53]. MGF files were searched using the most recent protein sequence libraries available from UniProtKB using X!Tandem [54] and OMSSA [55]. XML output files were parsed and non-redundant protein sets determined using Proteome Cluster based on previously published rules [56]. MS1-based isotopoic features were detected and peptide peak areas were calculated using the FeatureFinderCentroid tool, part of the OpenMS framework [52]. Proteins were required to have 1 or more unique peptides across the analyzed samples with E-value scores of 0.0001 or less.

### Statistical analyses

Geometric means (GM) were used as measures of central tendency. Data were analyzed for larval growth by multifactorial analysis of variance ANOVA with post-hoc Fisher’s Least Significant Difference (LSD) testing in Systat v.11 (Systat Inc., Evanstown, IL, USA). Probability values less than 0.05 were considered statistically significant. All experiments were performed a minimum of 2 times.

## Results

### Susceptibility of collaborative cross mice to infection with *O*. *volvulus* L3

To determine if there was an underlying genetic basis for the resistance of mice to infection with *O*. *volvulus* [14], 5 mouse strains having a wide range of genetic diversity from the CC (Cast/EIJ, IL16680, AU8052, AU8049 and OR13067) [57] were screened for their susceptibility to *O*. *volvulus*. Five mice from each of the different strains were infected with 100 *O*. *volvulus* L3 and necropsied at 4-weeks post infection. None of the 5 strains of CC mice tested was susceptible to infection with *O*. *volvulus* L3.

### Susceptibility of NSG mice to infection with *O*. *volvulus* L3

To assess the role of the mouse immune system in mediating resistance to *O*. *volvulus*, immunodeficient mouse strains were assessed for their susceptibility to infection with *O*. *volvulus*. In a preliminary set of studies, 250 *O*. *volvulus* L3 were injected into 2 NSG mice. After 4-weeks the skin and muscle from the mice was divided into 100 anatomically distinct sections and qPCR for O-150 was performed on extracted DNA from each section. Twenty-three of the sections were positive from, spatially widespread regions of the body. It was concluded that parasites survived in NSG mice and had the ability to migrate extensively. This indicated that all regions of the mice had to be inspected for the presence of parasites following infection.

**Fig 1:**
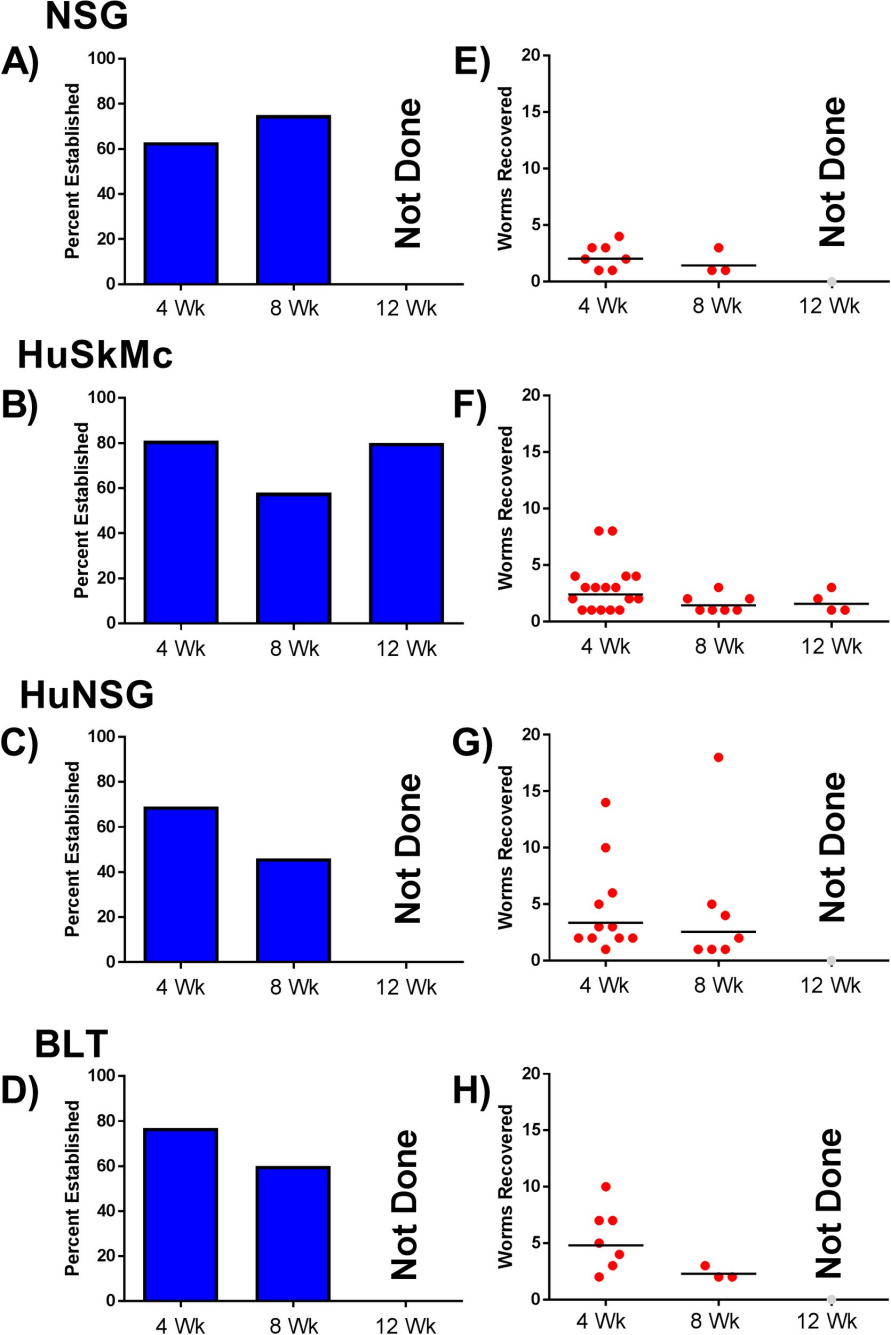
Survival of larval *Onchocerca volvulus* in four mouse models. *O*. *volvulus* L3 (100/mouse) were injected subcutaneously into 4 mouse models: (A,E) NSG mice (4 week n = 11 [2.3% Recovery], 8 week n = 4 [1.7% Recovery]), (B,F) Human skeletal muscle cell engrafted NSG mice (HuSkMc) (4 week n = 21 [3.0% Recovery], 8 week n = 12 [1.6% Recovery], 12 week n = 6 [1.8% Recovery]) (C,G) Humanized NSG (HuNSG) mice: NSG mice that received transfer of human CD34^+^ stem cells (4 week n = 16 [4.5% Recovery], 8 week n = 15 [4.6% Recovery]), (D,H) BLT mice: NSG mice engrafted with human fetal liver derived CD34^+^ stem cells and fetal thymus and liver tissues (4 week n = 9 [5.4% Recovery], 8 week n = 5 [2.3% Recovery]). After 4-, 8- or 12-weeks the percent established (the proportion of mice in a group of infected animals from which live parasites were recovered) (Fig 1A–1D) and the geometric mean number of live worms recovered per mouse within the group was determined (Fig 1E–1H).

**Fig 2:**
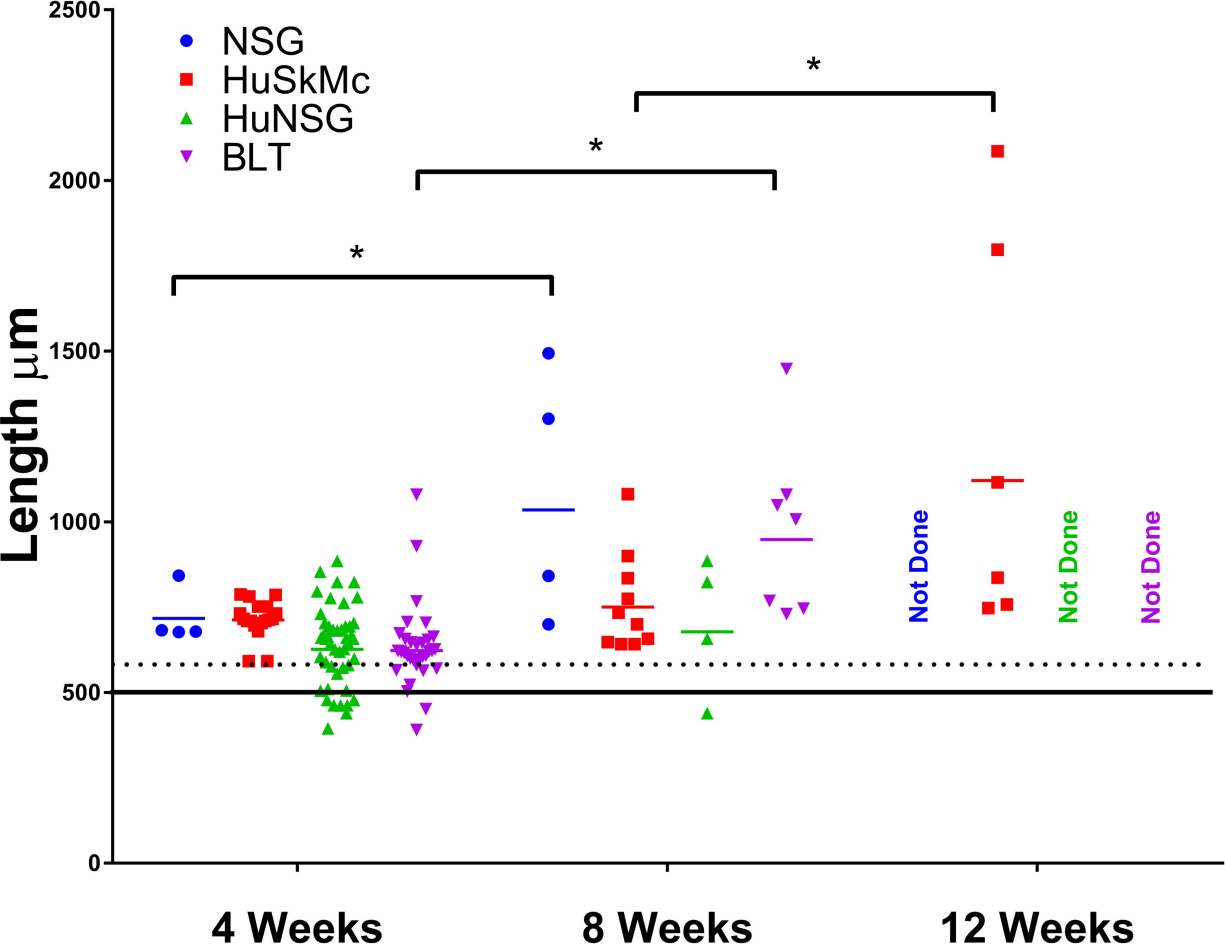
Growth of larval *Onchocerca volvulus* in four mouse models. O. *volvulus* L3 (100/mouse) were injected subcutaneously into 4 different NSG based models: (1) NSG, (2) Human skeletal muscle cell engrafted NSG mice (HuSkMc), (3) Humanized NSG (HuNSG) mice: NSG mice that had received a human CD34^+^ stem cell transfer, (4) BLT: NSG mice that had been engrafted with human fetal liver derived CD34^+^ stem cells and engrafted with fetal thymus and liver tissues. After 4, 8 or 12-weeks, animals were necropsied and worms were recovered and measured. Solid colored bar is the geometric mean of the lengths of larvae recovered. Solid black line is the geometric mean of the length of L3 recovered from black flies and dotted line is the 95^th^ confidence interval. *Asterisk represents statistical difference, p value ≤ 0.05, in length of larvae recovered from mice. Complete statistical analyses for all groups are included on S2 Table.

NSG mice were infected with 100 *O*. *volvulus* L3 and necropsied at 4- and 8-weeks following infection. Infected mice had an established infection rate of 63% with a GM worm recovery of 2.0 (range 1 to 4) worms recovered at 4-weeks following infection. At 8-weeks, 75% of mice had an established infection, with a GM recovery of 1.4 (range 1 to 3) worms per mouse (Fig 1A). The recovered worms were measured and found to be significantly increased in size (p<0.0001; Min: 677 μm, Max 843 μm, GM: 717 μm) at 4-weeks compared to L3. They also significantly increased in size between 4 and 8 weeks (p<0.0001) reaching a maximum of 1,085 μm (Min: 700 μm, GM: 1034 μm) (Fig 2).

### Susceptibility of NSG mice after receiving single human-cell xenografts

Because of the species-specific infectivity of *O*. *volvulus* (humans and primates), we next examined the ability of nutrients or growth factors from human cells to promote the growth and maturation of the parasite. Human cells were selected for study based on the anatomical niche they typically inhabit that would parallel the niches of *O*. *volvulus* in humans. Luciferin labeled HuSkMc cells injected into NSG mice had a life span of approximately one week based on *in vivo* imaging. Thus, NSG mice received weekly injections of BESM, LEC, HaCaT, or HuSkMc cells. Mice were injected with 100 *O*. *volvulus* L3 immediately after the cell inoculations. At 4-weeks post-inoculation of the L3, 60% of BESM cell engrafted mice established *O*. *volvulus* infection with a GM recovery of 1 worm per mouse with a maximum of 1 worm/mouse recovered (S1 Table). Mice engrafted with LEC had 20% established infection and GM of 4 worms recovered per mouse with a range of 1 to 4 worms per mouse (S1 Table). HaCaT cell engrafted mice had a 60% established infection and GM recovery 1.7 worms recovered per mouse with between 1 and 2 worms per mouse (S1 Table). HuSkMc cell engrafted mice had an 81% established *O*. *volvulus* infection rate at 4-weeks post-infection and GM recovery 2.4 worms recovered per mouse with between 1 and 8 per mouse. Based on the enhanced survival of *O*. *volvulus* in mice engrafted with HuSkMc cells, extended infections were evaluated. At 8-weeks post-infection, mice with HuSkMc cells had a 58% established infection rate with a GM parasite recovery of 1.4 with a range of 1 to 3 per mouse. At 12-weeks post-infection, 80% of the mice had established infections at recovery with a GM parasite recovery of 1.6 with 1 to 3 per mouse (Fig 1B). At each time point tested the parasites in HuSkMc-engrafted mice demonstrated continued parasite growth. At 4-weeks recovered parasites had GM lengths of 713 μm (Min: 592 μm, Max: 788 μm), at 8-weeks lengths of 751 μm (Min: 642 μm, Max: 1,081 μm), and at 12-weeks the GM length was 1,121 μm with a maximum length of 2,086 μm observed (Min: 748 μm), representing a 4-fold increase in length over L3 (Fig 2). These growth rates represented significant changes between L3 and 4-week worms (p = 0.0010) and between 8- and 12-week worms (p<0.0001).

### Susceptibility of NSG mice hosting multiple human cell types

SGM, NRG and NSG (HuNSG) mice humanized with CD34^+^ umbilical cord derived stem cells were infected with 100 *O*. *volvulus* L3. Each of these humanized mice had human hematopoietic lineage cells at a concentration greater than 600 cells per μl of blood. While a complete picture of the cell populations in these specific mice was not determined, based upon flow analysis during the screening process all humanized mice had both human B and T-cells present in their blood. At 4-weeks post-infection humanized SGM mice contained a GM of 2.4 (range 1–7) worms/mouse, humanized NRG mice had a GM of 1.7 (range 1 to 4) worms/mouse (S1 Table). HuNSG at 4-weeks post-infection had a 69% established infection rate with a GM of 3.4 (range 1–14) worms/mouse, and at 8-weeks post infection HuNSG mice had a 46% established infection rate and a GM of 2.6 (range 1–18) worms/mouse (Fig 1C). Larval growth in HuNSG mice was comparable to that seen in NSG and in NSG mice engrafted with HuSkMc (Fig 2). A significant increase in length was seen between the L3 and 4-week recovery (p<0.0001, Min: 395 μm, Max: 886 μm, GM: 627 μm), but no significant changes were seen between 4- and 8-weeks (Min: 440 μm, Max: 886 μm, GM: 678 μm) following infection.

BLT mice humanized with fetal thymus, liver and CD34^+^ stem cells, which display an enhanced repertoire of the developing human cells [58], were infected with 100 *O*. *volvulus* L3 and then necropsied at 4- and 8-weeks post infection. At 4-weeks post infection BLT mice had an established infection rate of 77% with a GM of 4.8 (range 2–10) worms/mouse. At 8-weeks post infection BLT mice had an established infection rate of 60% with a GM of 2.3 (range 2–3) worms/mouse (Fig 1D). Growth of the parasites was equivalent to that seen in the NSG mice and the other humanized models with the maximum length reaching 1,080 μm at 4-weeks (Min: 391 μm, GM: 623 μm) and 1,448 μm at 8-weeks (Min: 730 μm, GM: 949 μm) (Fig 2). The overall growth was significant between the L3 and worms 4-weeks post infection (p<0.0001) and between worms recovered at the 4- and 8-week time points (p<0.0001).

### Localization of parasites within the murine host

During necropsy, mice were sectioned into 4 groupings: upper muscles, lower muscles, skin, and the complete set of internal organs. No nodules were found in any of these tissues upon necropsy. Worms were recovered from all four of the different tissue groupings with no apparent preference for any region of the animal in all of the mice tested.

### Morphology

Detailed morphological analyses focused on worms recovered from BLT mice infected for 8-weeks and HuSkMc humanized mice infected for 12-weeks. Although there were clear differences in lengths of these worms, their morphological characteristics were similar. No differences in the ratio of males and female worms recovered from the mice was observed, however it was noted that most of the longer worms were females.

**Fig 3:**
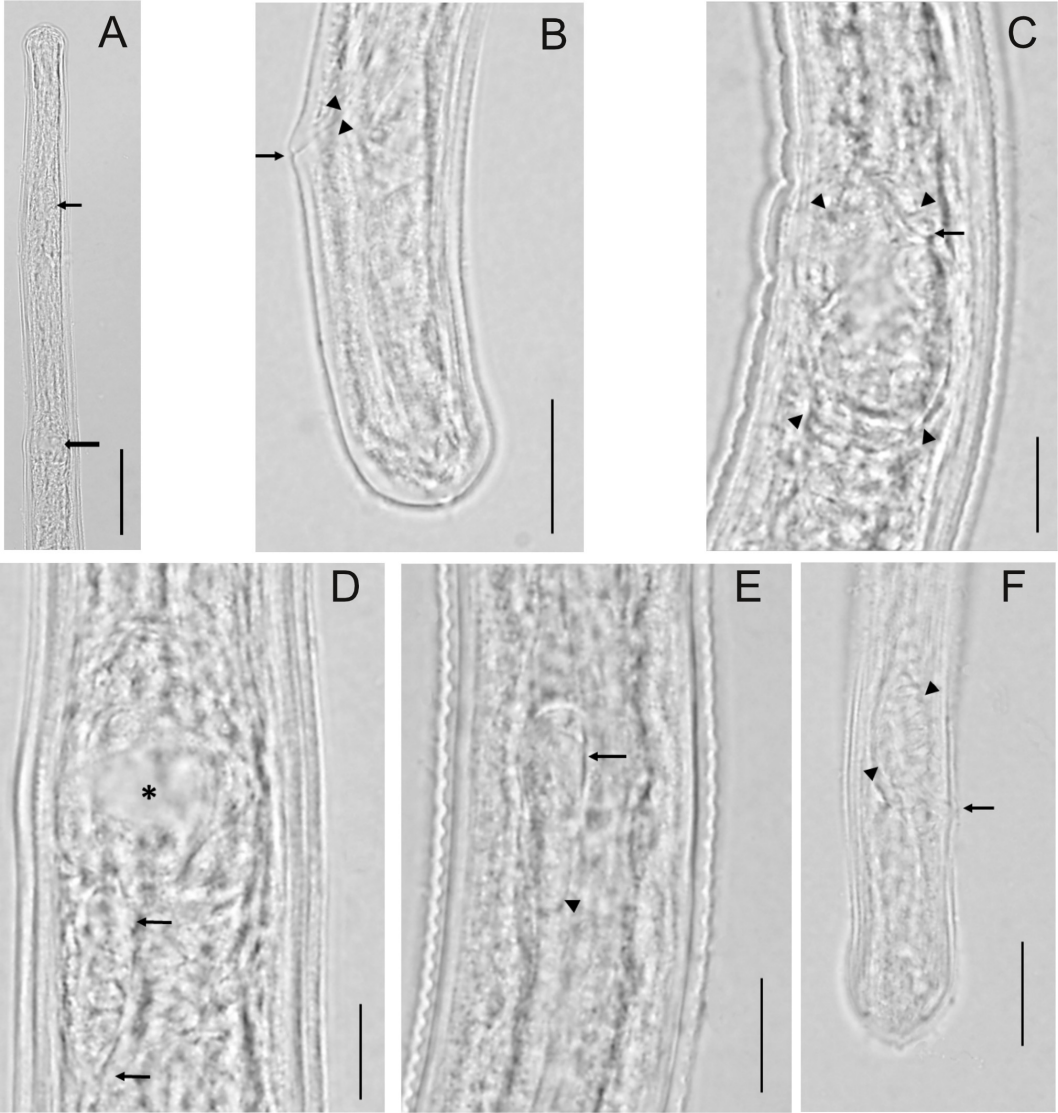
Imaging of *O*. *volvulus* larvae recovered from various models. A) Anterior end of female larva showing overall shape, location of nerve ring (small arrow) and vulva (large arrow). [Scale bar = 50 μm] B) Female tail, lateral view, showing overall shape and rectum (arrow heads) and anal opening (arrow). [Scale bar = 15 μm] C) Developing ovejector (arrowheads), lateral view, showing attachment to body wall and beginning of vulva (arrow). [Scale bar = 10 μm] D) Developing ovejector, dorsal-ventral view, showing the relative large cavity (asterisk) and the developing tube (arrows). [Scale bar = 10 μm] E) Male larva at approximately mid-body showing the testis (arrow), which is C-shaped, curved posteriorly, and has begun to grow posteriorly (arrowhead). [Scale bar = 10 μm] F) Male tail, lateral view showing overall shape. One developing spicule pad (arrowheads) is clearly visible as is the anal opening (arrow). [Scale bar = 15 μm].

Both the anterior and posterior ends in both sexes were bluntly rounded and only slightly tapered (Fig 3A, 3B and 3F). Other than growth in length, the major change from L3 was development of the reproductive systems. In the L3, both the female and male systems are rudimentary genital primordia consisting of only several cells. In the 8-12-week old worms, the female ovejector had formed and had attached to the body wall. The ovejector was ovoid in shape, relatively large and filled the body cavity, and had a distinct lumen (Fig 3C and 3D). Rudimentary cellular growth of the reproductive tubes was also evident (Fig 3D). In males, the testis, located at approximately mid-body, had become elongate in shape and had looped posteriorly to form a classic shepherd’s crook (Fig 3E). In addition, the spicule pads were well developed and demarcated (Fig 3F) but were still oval in shape and had not yet started to take on the shape of the spicules nor was there any evidence of cuticularization. These observations are consistent with parasite development into advanced fourth stage larvae (L4).

### Analysis of parasite biomarkers detected in serum and urine from infected mice

**Fig 4:**
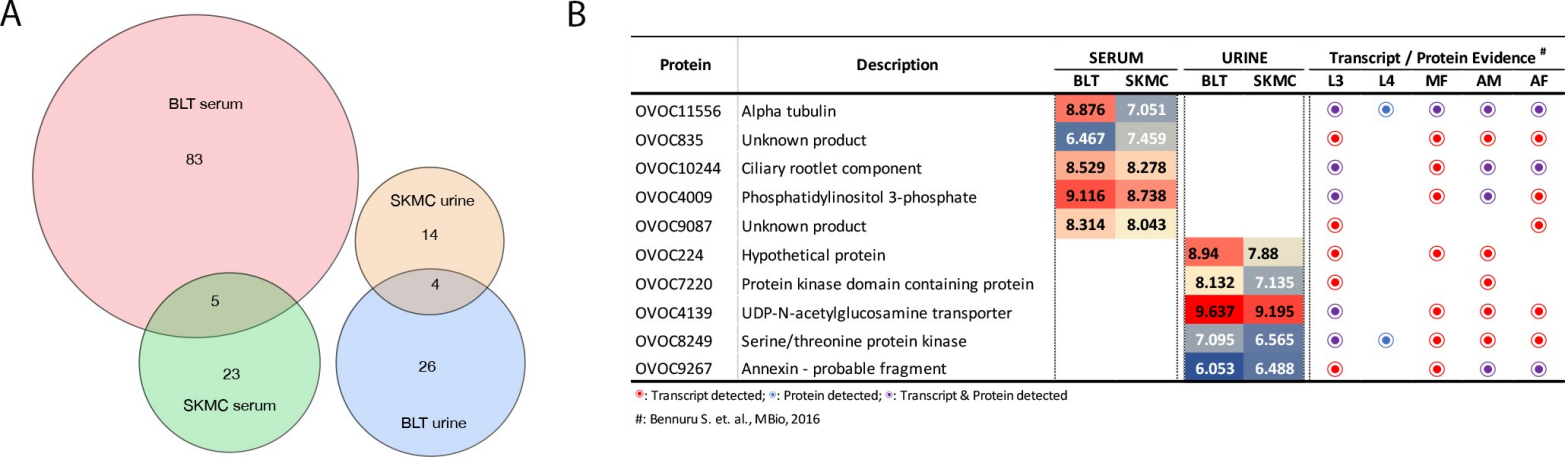
Proteomic identification in serum and urine of infected mice. A) Proportional venn diagrams showing the distribution of *O*. *volvulus*-proteins in the serum and urine of BLT (8-weeks) and HuSkMc (12-weeks) mice infected with *O*. *volvulus* L3. B) Proteins identified commonly in serum and urine shown in A. The buttons indicate evidence of the protein as transcript only (red), protein only (blue) or both transcript and protein (purple), across the stages.

Global proteomic analyses were performed with serum and urine collected from BLT mice infected for 8-weeks and HuSkMc humanized mice infected for 12-weeks. A total of 7,430 proteins were identified based on the spectral matching to a combined protein database of human, mouse, *O*. *volvulus* and its *Wolbachia* (*wOv*) endosymbiont. Because of the ambiguity in distinguishing certain spectral matches for proteins commonly found in both humans and mice, these proteins were grouped as non-*O*. *volvulus* proteins (4,743 in serum, 2,836 in urine, S1 Fig). The present study, however, focused on only *O*. *volvulus* derived proteins as potential parasite-encoded biomarkers of *O*. *volvulus* infection. Of all the proteins identified, 155 *O*. *volvulus* proteins (111 in serum and 44 in the urine) were detected in the infected mice and not in the control mice. While there were no proteins that were commonly detected in the serum and urine of the infected mice, the BLT and HuSkMc mice had 5 proteins (OVOC11556, OVOC835, OVOC10244, OVOC4009 and OVOC9087) in common in the serum and 5 (OVOC7220, OVOC4139, OVOC224, OVOC8249 and OVOC9267) in the urine (Fig 4B). Almost all the proteins identified have been shown through RNAseq to be transcribed by various stages of the *O*. *volvulus* parasite (Fig 4B).

## Discussion

The objective of this project was to identify small animal models that would support the development of black-fly derived *O*. *volvulus* L3 into advanced mammalian-adapted stages of the parasite. These small animal models are critically needed for identifying biomarkers released from the early stages of the infection and for screening potential new anthelmintics. Published findings on the susceptibility of mice to infection with the L3 of *O*. *volvulus* suggest that mice are resistant to infection when the larvae were injected subcutaneously [14]. However, *O*. *volvulus* L3 have been shown to survive and develop in mice when implanted within diffusion chambers at rates comparable to those seen in susceptible primates [17].

Genetic traits can play a significant role in the susceptibility of animals to infection as has been clearly demonstrated by the diversity of susceptibility of different mouse strains to infection with *Litomosoides sigmodontis* [59]. The CC mouse project was developed to produce an extremely diverse set of inbred animals that could be used for mapping different genetic traits [57]. Five different CC mouse strains, selected based on their diverse genetic backgrounds, were tested for susceptibility to *O*. *volvulus* and were completely resistant to the infection. This observation suggests that either mice are missing some integral factors required for parasite growth or the immune responses in mice are effective at eliminating the infection. The question remains as to why parasites are recovered live from diffusion chambers implanted in mice but not when injected into the tissues. There are several possible explanations including: (1) the diffusion chamber acts as a barrier from the immune response creating an immune privileged site, (2) the larvae within the diffusion chamber are blocked from migrating through the tissue releasing excretory and secretory products and thereby eliciting an immune reaction, or (3) the diffusion chamber attracts host components to the parasite microenvironment that are beneficial for parasite development.

To test the hypothesis that mouse-intrinsic immune responses control *O*. *volvulus* infections, NSG mice that lack both functional innate and adaptive immune systems were infected with *O*. *volvulus* L3. Advanced stages of the parasites were consistently recovered from the infected NSG mice, and parasites survived and developed over the 8-week time course into advanced L4. These findings demonstrate that the mouse immune response was capable of controlling infection with *O*. *volvulus*, with elements of the mouse immune response eliminating the infection in immunologically intact mice. Many mechanisms have been described for innate immune control of nematode infections in mice [39,60–67] all or some of which may be effective against *O*. *volvulus*. Interestingly, tissues and cells in NSG mice provide required factors for parasite development and based on PCR analyses, the larvae actively migrated far from the infection site.

The present study did not identify the point at which *O*. *volvulus* ceased surviving and developing in NSG mice. Studies with the human parasite *S*. *stercoralis* have demonstrated that the entire parasite life cycle will develop in NSG mice within 4-weeks [31]. Given the significant difference in time that it takes for *O*. *volvulus* adults to develop (12–15 months in chimpanzees [9,10]) and their size as mature adults (females are 50 cm [68]) it is unlikely that the entire *O*. *volvulus* life cycle, including mating and development of microfilariae, will occur in NSG mice.

*In vitro* studies on the development of filarial worms including *O*. *volvulus* [69,70] and *Brugia malayi* [71] have demonstrated that host cells are needed in the culture wells to optimize parasite growth and development. Furthermore, optimal development and survival of larval *T*. *spiralis* in mice requires the presence of mouse eosinophils [33]. It was thus hypothesized that adding human cells to the NSG mice, from the tissues that the parasites are normally found juxtaposed in humans, might provide additional required nutritional or developmental elements found in humans required for parasite development and survival. Four different single cell xenografts were screened: HuSkMc, LEC, HaCaT and BESM. Of these four cell lines, HuSkMc was found to support the highest average percent survival of the implanted worms and consistent infection rates over a 12-week time period (Figs 1 and 2)

As an alternative to adding single cell populations to the NSG mice, multipotential umbilical cord stem cells were transferred to the immunodeficient mice. NSG, NSG-SGM, and NRG mice, all of which lack functional immune responses, were humanized with CD34^+^ umbilical cord stem cells. The humanization of the various immunocompromised mouse strains resulted in the development of an immature human immune system. The developing immune system in these mice displays a T-independent response, limited antigen-specific IgM responses, and the presence of multiple innate immune cells has been noted [25]. When humanized NSG, NSG-SGM, and NRG mice were infected with *O*. *volvulus* L3, higher GM parasite recoveries were observed when compared to NSG mice without any human cells but these enhancements were not significant. Although NSG-SGM did have consistent infection rates with *O*. *volvulus* we had significant difficulties establishing reliable engraftment of human cells in this strain of mice. Extending this concept further, BLT mice which contain CD34^+^ stem cells in addition to fetal tissue were used. BLT mice are known to develop a more mature version of a human immune system. Limited T-dependent recognition is seen within these mice and better overall functionality of both the B and T-cells has been documented [36]. BLT mice supported the highest average parasite recoveries of any mouse model tested at 4-weeks, although the average fell to be in line with the HuNSG and HuSkMc models by 8-weeks post infection. The mean lengths of the parasites recovered from 4- and 8-week time points from the NSG mice were comparable to those recovered from BLT mice. This suggests a link between the enhanced survival, may be related to the presence of the human immune cells, but the growth of the parasites that survive may not be related to the human cell byproducts. Although cellular engraftments levels in BLT and HuNSG mice were not rechecked at the end of the experiment previously published data have shown that these animals reliably hold their engraftments for extended periods of time [72,73].

The mouse models developed in this study offer a number of advantages over the models that currently exist. L3 implanted within diffusion chambers in both non-human primates and mice successfully molt into L4 but the lengths of the recovered parasites were significantly shorter than those recovered at 8 and 12 weeks post infection from the mice tested in this study [17]. While non-human primates can support the development of the parasites from L3 to microfilariae producing adults, cost and ethical considerations limit the utility of primates for drug discovery and antigen identification [10,14]. Nodules containing adult worms recovered from humans have been implanted into SCID mice and the female worms survived and released microfilariae [15]. This method allows for identification of adult antigens and biomarkers, but lacks the intermediate L3 and L4 stages of development. Finally, an alternative model using adult male *O*. *ochengi* implanted into SCID mice has been developed for the identifying filaricides [16], which may also be effective against *O*. *volvulus*. The NSG models developed in this study have the critical advantage of working with *O*. *volvulus*, thereby allowing species-specific screening of filaricides and identification of biomarkers.

It was of interest to note that the immune response found in normal mice was highly efficient at eliminating the L3 of *O*. *volvulus*, but the human immune cells in NSG mice were supportive rather than destructive of the parasites. It is possible that the mouse immune cells were evolutionally adapted to eliminate the parasites, whereas the human cells were evolutionally adapted to support the parasites as observed in nature. A comparison between mouse and human immune reactions to the worms might yield important new insights into the etiology of resistance and susceptibility to infection with *O*. *volvulus*.

The source of the L3 used in these studies was from parasites cryopreserved in liquid nitrogen. After defrosting, 100 individual parasites were selected, attempting to identify only the viable/undamaged L3. It is reasonable to predict that a percentage of larvae exiting from the mouth parts of a black fly have the potential to resume development in the human host and that the cryopreservation process damaged some of those worms. Approximately 50 percent of the larvae recovered after cryopreservation survived in diffusion chambers implanted in mice, which may explain the overall number of worms recovered from the different mouse models in this study. While minor differences in the overall larval recovery levels were observed between individual batches of cryopreserved larvae, multiple batches were combined before implantation to help ensure a consistent viability level going into the animal hosts. Even with the combination of multiple batches of larvae it cannot be ruled out that the overall viability of the injected larvae played a role in the observed recovery rates. The recovery rates of *O*. *volvulus* larvae after developing in NSG mice with or without human cells was in the same order of magnitude as that reported for the recovery of adult filarial worms, where infections were initiated by larvae recovered directly from the insect vector. These infections included *Brugia pahangi* in cats [74], *Brugia malayi* in leaf-monkey [75], and *Onchocerca ochengi* in cattle [76]. In the final analysis, optimal parasite recovery was observed in mice humanized with HuSkMc (maximum of 10 worms), HuNSG (maximum of 18 worms) and BLT mice (maximum of 10 worms).

It was clear that worms increased in size during the infection period with individual worms achieving up to 4 times the size of the original L3. Growth of worms within a single mouse was not consistent and suggests that there is significant variability within the infecting larval population. It does verify, however, that humanized mice have the potential to support extended development of *O*. *volvulus*. Both male and female worms grew in length and resumed their sexual development. Although no cast cuticles were observed, it was evident from organ development that the parasites had molted into fourth-stage larvae.

Urine and serum was collected from humanized mice infected for 8-12-weeks with *O*. *volvulus* for the identification of biomarkers. Infected HuSkMc mice or BLT mice were selected for this analysis so the biomarkers identified would develop in the presence of human cells thereby potentially enhancing their specificity. Several *O*. *volvulus*-specific peptides were identified in the serum and urine of BLT and HuSkMc mice (S3 Table), however, no *O*. *volvulus*-specific proteins were found in both urine and serum from either mouse source. The most likely useful biomarkers were the proteins listed in Fig 4B. Though most of the proteins were identified by one or more unique peptide(s), among the proteins with unknown function (OVOC9087, OVOC835, OVOC224), OVOC9087 does not have orthologues in other filarial species and hence would likely be able to distinguish *O*. *volvulus* from other filarial infections. Because of the expected low number and abundance of *O*. *volvulus*-specific protein identification in the serum and urine from any given mouse in the current system, mass spectrometry was carried out with pooled serum and urine samples from each group of mice.

In conclusion, novel small-animal hosts, NSG mice, have been identified that support the survival and development of *O*. *volvulus* L3 into advanced L4 mammalian stages. Humanized mice have also been shown to be effective at identifying biomarkers for early *O*. *volvulus* infections. It is anticipated that these small-animal hosts for *O*. *volvulus* will also be useful as part of the effort to identify new anthelminthic drugs. Finally, the fact that *O*. *volvulus* survives and develops in NSG mice humanized with human immune cells may provide the opportunity to study the human immune response to early infection with *O*. *volvulus* in a small animal model.

## Supporting information

S1 Fig Proteomic identifications of non-*O*. *volvulus* derived proteins: Proportional Venn diagrams depict the number of proteins identified in the (A) serum and (B) urine of control mice and BLT, HuSkMc mice infected with *O*. *volvulus* L3 larvae.
(TIF)

S1 Table Survival of larval *Onchocerca volvulus* in five mouse models. NSG were engrafted with the following single cell types: LEC, HaCaT, or BESM. In addition NRG (HuNRG) or SGM (HuSGM) mice were engrafted with human cord blood CD34^+^ stem cells. Mice were infected with 100 *O*. *volvulus* L3 and 4 weeks post infection mice were necropsied and worms recovered. Percent established refers to the proportion of mice in a group of infected animals from which live parasites were recovered. Numbers displayed are the geometric mean and range of live worms recovered per mouse.
(XLS)

S2 Table Statistical analyses comparing parasite lengths recovered from NSG, Human Skeletal Muscle Cell engrafted NSG Mice (HuSkMc), CD34^+^ cord blood stem cell engrafted NSG mice (HuNSG), and CD34^+^ fetal liver stem cell engrafted NSG mice containing human fetal thymus and liver tissues (BLT). Data were analyzed for parasite growth by multifactorial analysis of variance ANOVA with post-hoc Fisher’s Least Significant Difference (LSD) testing. A) Comparison between infective L3 and worms recovered from NSG, HuSkMc, HuNSG, and BLT 4 weeks post infection, B) Comparison between infective L3 and worms recovered from NSG, HuSkMc, HuNSG, and BLT 8 weeks post infection. C) Comparison between infective L3 and worms recovered from NSG at 4 and 8 weeks post infection. D) Comparison between infective L3 and worms recovered from HuSkMc at 4, 8, and 12 weeks post infection. E) Comparison between infective L3 and HuNSG at 4 and 8 weeks post infection. F) Comparison between infective L3 and worms recovered from BLT at 4 and 8 weeks post infection.
(XLSX)

S3 Table All *O*. *volvulus*-derived proteins identified in the serum and urine infected mice. The table lists all the *O*. *volvulus*-derived proteins identified exclusively in the infected mice and not in the control mice. The values denote peak area intensity of the peptides identified for each protein.
(XLSX)
