# Supplementary figures and images for: Development of Onchocerca volvulus in humanized NSG mice and detection of parasite biomarkers in urine and serum
Source: PLoS Negl Trop Dis. 2018 Dec 12;12(12):e0006977. doi: 10.1371/journal.pntd.0006977 (PMC6306240; doi:10.1371/journal.pntd.0006977)

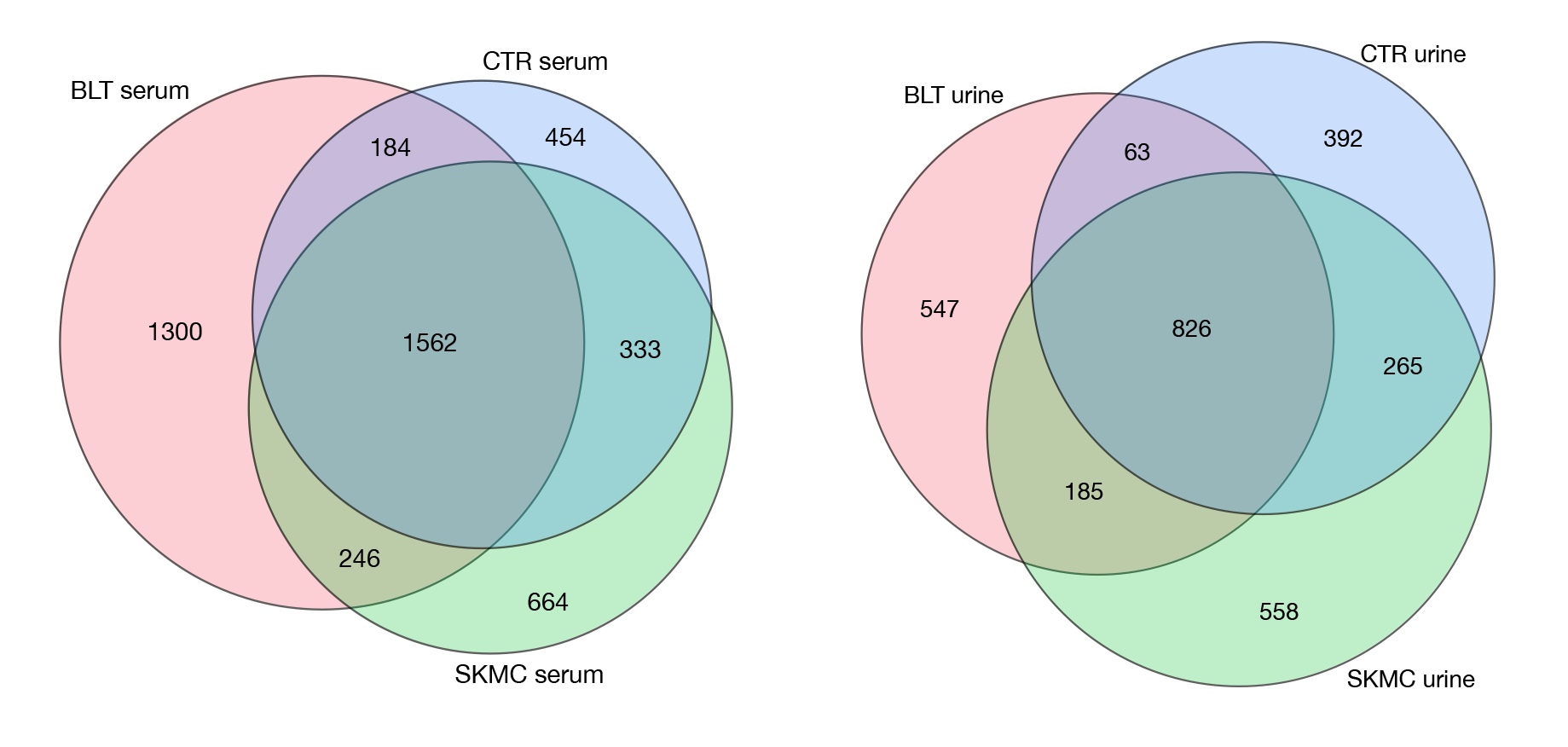

Supplement: S1 Fig — Proteomic identifications of non-O. volvulus derived proteins: Proportional Venn diagrams depict the number of proteins identified in the (A) serum and (B) urine of control mice and BLT, HuSkMc mice infected with O. volvulus L3 larvae. (TIF) [file pntd.0006977.s001.tif]
